# Early vulvar and umbilical incisional scar recurrence of cervical squamous cell carcinoma: Earlier than usually expected

**DOI:** 10.4274/tjod.40225

**Published:** 2017-06-15

**Authors:** Hüseyin Çağlayan Özcan, Aynur Mustafa, Zehra Bozdağ, Seyhun Sucu, Mete Gürol Uğur, Özcan Balat

**Affiliations:** 1 Gaziantep University Faculty of Medicine, Department of Obstetrics and Gynecology, Gaziantep, Turkey; 2 Gaziantep University Faculty of Medicine, Department of Pathology, Gaziantep, Turkey

**Keywords:** Squamous cell cervical cancer, umbilical metastasis, vulvar metastasis

## Abstract

Cutaneous metastasis is considered as a hazardous condition depending on the mean survival around 9 months, which usually originates from cancers of the breast, lung, ovary, colon, and rarely from the cervix. The crucial prognostic factor of cutaneous metastasis depends on the period between the primary malignancy and cutaneous metastasis. We report two cases of the unusual presentation of squamous cell cancer of the cervix that developed vulvar and umbilical metastasis in the 5^th^ month of primary treatment. Both of our patients survived for 11 months following the primary treatment. In addition, our first case is the earliest vulvar recurrence of cervical carcinoma in the English literature following appropriate medical and surgical management.

## INTRODUCTION

Cervical cancer recurrence depends on the cancer’s clinical stage and may manifest as local or distant metastasis in different organs. Recurrence occurs most commonly in the pelvis, which includes the parametrium or lymph nodes, and in the vagina. Recurrence can rarely occur in the skin, ranging between 0.1-1.3%. In most cases, they manifest as an asymptomatic dermal/subcutaneous plaque, ulcer or nodule^(1)^.

We report two unusual presentations of cervical squamous cell carcinoma with early vulvar and umbilical metastasis.

## CASE REPORTS

### Case 1

**Figure 1:**
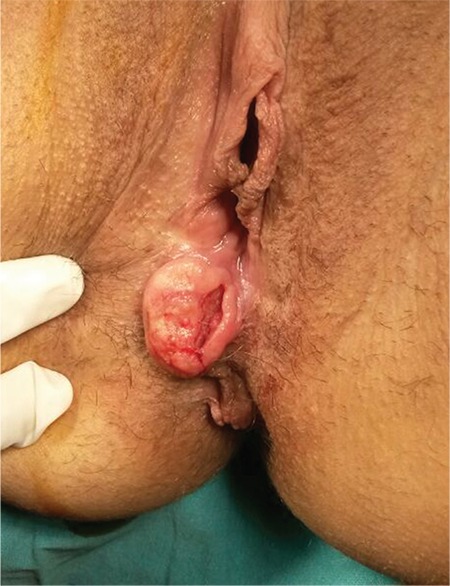
A 2x3-cm ulcerated fragile, firm, nodular lesion with irregular boundaries on the right labium majus with focal central hemorrhage

**Figure 2:**
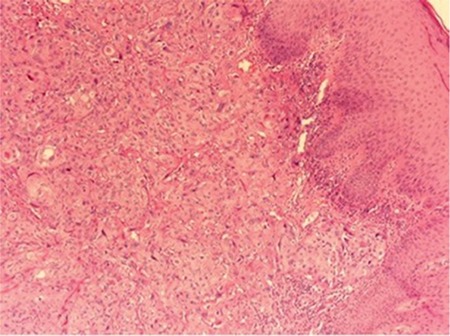
Infiltrating tumor nests consisting of atypical squamous cells with large abundant eosinophilic cytoplasm and a large vesicular nucleus with prominent nucleoli

A woman aged 41 years was admitted to a state hospital with pelvic pain, urinary burning, and vaginal bleeding. The patient was referred to our hospital after a cervical biopsy revealed cervical squamous cell carcinoma. On our physical examination, we observed an exophytic necrotic mass measuring 8x9 cm confined to the cervix with no parametrial invasion. According to the International Federation of Gynecology and Obstetrics classification, we established the diagnosis as stage 1b-2 cervical cancer. We excised the mass through the vagina route and performed a type 3 radical hysterectomy (Wertheim) with pelvic-paraaortic lymph node dissection followed by radiotherapy. In the fifth month after surgical treatment, we observed a 2x3-cm ulcerated nodular vulvar lesion (Figure 1). The lesion in the vulva was excised following abdominopelvic computerized tomography (CT) imaging, which revealed no significant pathology. The biopsy specimen showed squamous cell carcinoma (Figure 2). A paclitaxel and carboplatin combined chemotherapy protocol was used. Following 2 cycles of chemotherapy, positron emission tomography-CT revealed diffuse metastases in the abdominopelvic site. Despite and alternative protocol (gemcitabine and bevacizumab) administration, there was no response. The patient died in the 11^th^ month of the postoperative period.

### Case 2

**Figure 3:**
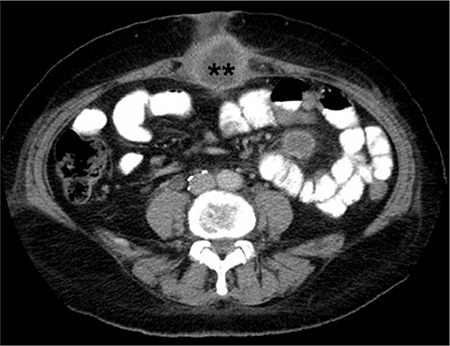
Computerized tomography image of metastatic umbilical mass

A woman aged 54 years who was post-menopausal presented with vaginal bleeding that had persisted for 3 months. A vaginal examination revealed a cervical mass measuring 1x1.5 cm. Histologic examination of the mass showed cervical squamous cell carcinoma. A Wertheim operation was performed and there was no lymph node involvement and the mass had negative surgical borders (stage 1b-1). A Papanicolaou smear was obtained from the vaginal cuff 3 months later and the result was negative. The patient presented with severe abdominal pain, which was localized along the incisional scar region of her umbilicus five months after the primary surgical treatment. Abdominal CT revealed an umbilical mass measuring 4x4.5 cm in diameter (Figure 3). We considered that the fixed mass was inoperable; it included all layers of the umbilical wall and extended from umbilicus to the upper anterior abdominal wall with massive adhesions. We performed a partial resection of the mass and pathologic examination revealed metastatic squamous cell carcinoma. Two cycles of chemotherapy, including paclitaxel-carboplatin in the first cycle and bevacizumab-gemcitabine in the second cycle, and radiotherapy was administered. The patient died in the 11th month of her medication.

## DISCUSSION

Cutaneous metastasis usually originates from cancers of the breast, lung, ovary, colon, and rarely from the cervix. Cervical carcinoma metastases frequently occur in the vulva and anterior abdominal wall or scalp, extremities, and the umbilical surgical scar can be affected, albeit rarely^(1)^. Invasive interventions, including paracentesis, laparoscopy, and laparotomy can also play a role in metastases of the cervix^(2)^. In addition, cutaneous metastases have an incidence of 0.8% in treated cervical cancers^(3)^. Adenocarcinoma and undifferentiated carcinoma of the cervix are the primary histopathologic types that contribute to cutaneous metastasis. However, there is no correlation between its prevalence and clinical stages^(4)^.

Cervical carcinoma can spread either locally or through lymphatic vessels. The lymphatic route usually follows pelvic, paraaortic and/or supraclavicular nodes, respectively. Cervical lymphatics are drained through pre-ureteral, post-ureteral, and uterosacral nodes, but these routes cannot clarify vulvar involvement. Vaginal-vulvar pathways and hematogenous invasion could be the possible routes of vulvar and umbilical incisional scar invasions, respectively. These theories have not been proven by either histologic or imaging methods. Tumor invasion to pelvic organs and the vulva can be explained by the close anatomic relationship^(5)^. Patients with cervical cancer metastasis can present with different symptoms. In our cases, painless skin lesions and severe abdominal pain localized in the umbilicus were the first signs of metastases during follow-up. It is very rare to detect cutaneous vulvar metastasis originating from cervical cancer before between 3.5 and 6 years after surgery^(2,4)^. However, this is the earliest vulvar metastasis (in the 5^th^ month of primary treatment) of cervical cancer in the English literature. A review of the relevant literature concerning vulvar metastasis is summarized in Table 1. In contrary, there are some reports regarding early umbilical recurrence of cervical cancer in the 4^th^, 5^th^, and 6^th^ months of primary treatment^(2,8,9)^. There are approximately 17 reports regarding umbilical metastasis of cervical cancer in the literature^(2,10,11,12,13,14)^.

Cutaneous metastasis is considered as a hazardous condition; the mean life span is around 9 months. Regarding one study that included 1190 patients with cervical carcinoma, the incidence skin metastasis was 1.3%, which increased with advanced clinical stage as follows: 0.8% in stage 1, 1.2% in stages 2 and 3, and increasing to 4.8% in stage 4. The presence of such metastasis is associated with a high mortality rate within 2 years, regardless of the treatment procedure^(15)^. Therefore, the crucial prognostic factor depends on the period between the primary malignancy and cutaneous metastasis. In other words, earlier metastasis means poorer prognosis. Survival was 11 months in both of our cases, most probably due to the short recurrence period (5^th^ month) following the primary surgical treatment.

Palliative surgery, chemotherapy, radiation therapy alone and/or in combination with cisplatin-based chemotherapy are well-known treatment modalities in managing advanced recurrent disease^(16)^. Based on that, we applied platinum-based chemotherapy in both cases and concurrent radiotherapy after detecting vulvar and umbilical scar extensions, respectively. Nevertheless, there was no response and we administered an alternative protocol (gemcitabine and bevacizumab) in both cases, which may have prolonged survival to up to approximately one year.

In conclusion, cervical cancer rarely leads to vulvar and umbilical incisional scar metastasis, which can be accepted as a poor prognostic factor accompanied with short life span. Physicians should always keep in mind the likelihood of recurrence at these locations during follow-up in cases of cervical cancer. To the best of our knowledge, our first case is the earliest vulvar recurrence and our second case is one of the earliest recurrences of cervical carcinoma in the English literature following appropriate surgical and medical management.

## Figures and Tables

**Table 1:**
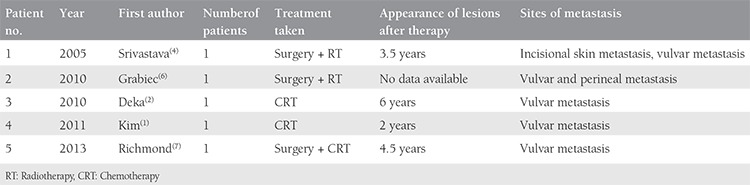
Reported cases of vulvar metastasis in cervical cancer
